# How Energy Metabolism Supports Cerebral Function: Insights from ^13^C Magnetic Resonance Studies *In vivo*

**DOI:** 10.3389/fnins.2017.00288

**Published:** 2017-05-26

**Authors:** Sarah Sonnay, Rolf Gruetter, João M. N. Duarte

**Affiliations:** ^1^Laboratory for Functional and Metabolic Imaging, École Polytechnique Fédérale de LausanneLausanne, Switzerland; ^2^Department of Radiology, University of LausanneLausanne, Switzerland; ^3^Department of Radiology, University of GenevaGeneva, Switzerland

**Keywords:** brain energy metabolism, neurotransmitter metabolism, neuron-glia interaction, neuronal activity, MRS, fMRI, mathematical modeling

## Abstract

Cerebral function is associated with exceptionally high metabolic activity, and requires continuous supply of oxygen and nutrients from the blood stream. Since the mid-twentieth century the idea that brain energy metabolism is coupled to neuronal activity has emerged, and a number of studies supported this hypothesis. Moreover, brain energy metabolism was demonstrated to be compartmentalized in neurons and astrocytes, and astrocytic glycolysis was proposed to serve the energetic demands of glutamatergic activity. Shedding light on the role of astrocytes in brain metabolism, the earlier picture of astrocytes being restricted to a scaffold-associated function in the brain is now out of date. With the development and optimization of non-invasive techniques, such as nuclear magnetic resonance spectroscopy (MRS), several groups have worked on assessing cerebral metabolism *in vivo*. In this context, ^1^H MRS has allowed the measurements of energy metabolism-related compounds, whose concentrations can vary under different brain activation states. ^1^H-[^13^C] MRS, i.e., indirect detection of signals from ^13^C-coupled ^1^H, together with infusion of ^13^C-enriched glucose has provided insights into the coupling between neurotransmission and glucose oxidation. Although these techniques tackle the coupling between neuronal activity and metabolism, they lack chemical specificity and fail in providing information on neuronal and glial metabolic pathways underlying those processes. Currently, the improvement of detection modalities (i.e., direct detection of ^13^C isotopomers), the progress in building adequate mathematical models along with the increase in magnetic field strength now available render possible detailed compartmentalized metabolic flux characterization. In particular, direct ^13^C MRS offers more detailed dataset acquisitions and provides information on metabolic interactions between neurons and astrocytes, and their role in supporting neurotransmission. Here, we review state-of-the-art MR methods to study brain function and metabolism *in vivo*, and their contribution to the current understanding of how astrocytic energy metabolism supports glutamatergic activity and cerebral function. In this context, recent data suggests that astrocytic metabolism has been underestimated. Namely, the rate of oxidative metabolism in astrocytes is about half of that in neurons, and it can increase as much as the rate of neuronal metabolism in response to sensory stimulation.

## Introduction

Cerebral function requires the cooperative interaction between different cell types, namely neurons, astrocytes, microglia and oligodendrocytes, and depends on high metabolic activity supported by continuous supply of oxygen and glucose from the blood (Siesjö, 1978). Blood flow is indeed directly related to the cerebral metabolic rate of glucose consumption (CMR_glc_) (Sokoloff, 1978). Although the adult human brain represents only 2% of the total body weight, it consumes up to 20% of the total glucose metabolism under normal resting physiological conditions (e.g., Rolfe and Brown, 1997). Since the mid-twentieth century the idea that brain energy metabolism is coupled to neuronal activity has emerged (McIlwain et al., 1951; Van den Berg et al., 1969), and a number of studies supported this hypothesis (Pellerin and Magistretti, 1994; Poitry-Yamate et al., 1995; Tsacopoulos et al., 1997). Notably, in the 90's, brain energy metabolism was demonstrated to be compartmentalized between neurons and astrocytes, and astrocytic glycolysis was proposed to serve the energetic demands of glutamatergic activity (Pellerin and Magistretti, 1994; Poitry-Yamate et al., 1995; Tsacopoulos et al., 1997).

Ogawa et al. reported in 1992 the changes in the apparent transverse relaxation time ${\text{T}}_{2}^{*}$ due to variations in local blood oxygen consumption (CMR_O2_), cerebral blood flow (CBF) and cerebral blood volume (CBV; Ogawa et al., 1992). This discovery formed the basis of a powerful technique used nowadays to study brain activity: blood oxygenation level-dependent (BOLD) functional magnetic resonance imaging (fMRI). Under the assumption of brain metabolism being segregated into two main compartments, neurons and astrocytes (which is valid for cortical gray matter), and based on measurements of the glutamate-glutamine cycle and glucose oxidation rates, a quantitative interpretation of functional imaging by integrating oxidative neuroenergetics of neuronal processes was thereafter suggested (Shulman and Rothman, 1998). In this context, the main metabolic costs underlying neuronal activity involved not only the maintenance of the glutamate-glutamine cycle, but also the generation and propagation of action potentials, uptake and recycling of neurotransmitters from the synaptic cleft, and restoration and maintenance of resting membrane potential (reviewed in Attwell and Laughlin, 2001). However, besides the proposed coupling between neurotransmission and neuronal oxidative metabolism, data acquired during the past decades in other experimental conditions and models suggested substantial astrocytic contribution to metabolism (Gruetter et al., 2001 and reviewed in Lanz et al., 2013) and blood flow regulation (reviewed in Attwell et al., 2010). A recent analysis on K^+^-dependent stimulation of astrocytic metabolism suggests that the actual glial contribution to total energy metabolism has been long underestimated (DiNuzzo et al., 2017).

This article reviews the biochemical mechanisms associated with energy metabolism in brain cells, and provides a critical review of the traditional view of astrocytes being glycolytic and neurons oxidative, which has been challenged over the past years by evidence pointing to important rates of oxidative respiration in astrocytes, namely during increased brain activity. ^13^C MRS along with infusion of ^13^C-labeled substrates and the use of compartment models as tools to probe glial and neuronal metabolism will then be described. Data recently acquired in our laboratory (Sonnay et al., 2016, 2017) assessing the matter of glial and neuronal oxidative metabolism coupled to neuronal activity is then presented and potential usage of the mitochondrial ATP production in astrocytes is further discussed.

## Brain glucose uptake and metabolism

The brain can consume several substrates, such as lactate (Bouzier et al., 2000; Wyss et al., 2011), acetate (Cerdan et al., 1990), fatty acids (Kuge et al., 1995) and ketone bodies (Künnecke et al., 1993), but energy metabolism in the adult brain primarily relies on glucose provided from the blood to fuel activity both in the resting and activated states (reviewed in Sokoloff, 2004).

Uptake of monocarboxylates, such as lactate, pyruvate, and ketone bodies, is mediated by monocarboxylate transporters (MCT) along with the co-transport of one ^1^H for each molecule. The isoform MCT1 is expressed in the endothelial cells and in astrocytes (reviewed in Pierre and Pellerin, 2005), MCT4 in astrocytes and MCT2 in neurons (Bergersen et al., 2002; and reviewed in Barros and Deitmer, 2010).

In mammalian brain cells, glucose transport and utilization is predominantly mediated by facilitated diffusion through glucose transporters GLUT1 and GLUT3 that belong to the Solute Carrier Family 2 (SLC2). GLUT1 is present in all brain cells, with high density in astrocytes and endothelial cells of the capillaries, but less in neurons (reviewed in Maher et al., 1994). In contrast, GLUT3 expression is almost restricted to neurons (Maher et al., 1992, 1996). GLUT1 is thus the main carrier involved in the import of glucose into the brain from the blood, and its apparent affinity for glucose transport is lower than that of GLUT3 (discussed in Simpson et al., 2007). These two facilitative carriers mediate energy-independent transport of glucose bi-directionally along a concentration gradient, which is maintained by continuous phosphorylation of intracellular glucose by the glycolytic enzyme hexokinase, and exist in sufficient density to ensure that glucose transport is not rate-limiting for CMR_glc_ (Gruetter et al., 1998b; Barros et al., 2007; Duarte et al., 2009). GLUT4 in neurons (Ashrafi et al., 2017) and GLUT2 in both neurons and astrocytes (Thorens, 2015) have also been shown to transport glucose. However, GLUT2 and GLUT4 are carriers involved in specific functions in certain brain areas, and are likely to have a minor role on glucose uptake for cellular fueling.

After entering the cells, glucose is converted via glycolysis to two molecules of pyruvate with net formation of 2 ATP and 2 NADH in the cytosol. Pyruvate can then be reduced to lactate mediating NAD^+^ formation, transaminated to alanine or enter mitochondria via the mitochondrial pyruvate carrier, where it is decarboxylated to acetyl-CoA by the pyruvate dehydrogenase complex (PDH) with formation of CO_2_ and NADH (Patel and Korotchkina, 2001). Acetyl-CoA condensates with oxaloacetate entering therefore oxidative metabolism via the tricarboxylic (TCA) cycle. Each turn of the TCA cycle yields 3 NADH, 1 FADH_2_ and 1 GTP molecules. The electron-transfer chain generates a gradient of H^+^ across the mitochondrial membrane, which is used by the ATP synthase for ATP production. As each NADH and FADH_2_ molecules generates 2.5 and 1.5 ATP respectively, complete oxidation of one molecule of glucose produces 30 or 32 ATP, depending on the transport of cytosolic NADH to mitochondria either in the malate-aspartate or in the glycerol 3-phosphate mitochondrial shuttles (Voet and Voet, 1995).

Oxidation of glucose-derived pyruvate through the TCA cycle not only provides the bulk of energy produced to support cerebral function (reviewed in Hertz and Dienel, 2002), but also involves the generation of *de novo* amino acids, namely glutamate (reviewed in Gruetter, 2002). Neurons extensively release glutamate and need therefore a replenishment system to ensure adequate neurotransmitter levels. Namely, synthesis of *de novo* oxaloacetate from pyruvate is catalyzed by the glial-specific enzyme pyruvate carboxylase (PC; Gamberino et al., 1997), mediating CO_2_ fixation in an energy-dependent manner, increasing therefore the number of carbon skeletons in the TCA cycle. Oxaloacetate formed through pyruvate carboxylation condensates with acetyl-CoA to produce new glutamate molecules (Waagepetersen et al., 2001). In addition, under low acetyl-CoA concentration, pyruvate can be produced cataplerotically from TCA cycle intermediates (pyruvate recycling): from oxaloacetate, mediated by the combined action of phosphoenolpyruvate carboxykinase (PEPCK) and pyruvate kinase (PK; Cruz et al., 1998), occurring in astrocytes (Sonnewald et al., 1996) and to less extent in neurons (Cruz et al., 1998), and from malate by the malic enzyme (ME; Bakken et al., 1997; Cruz et al., 1998; Sonnewald, 2014).

**Figure 1:**
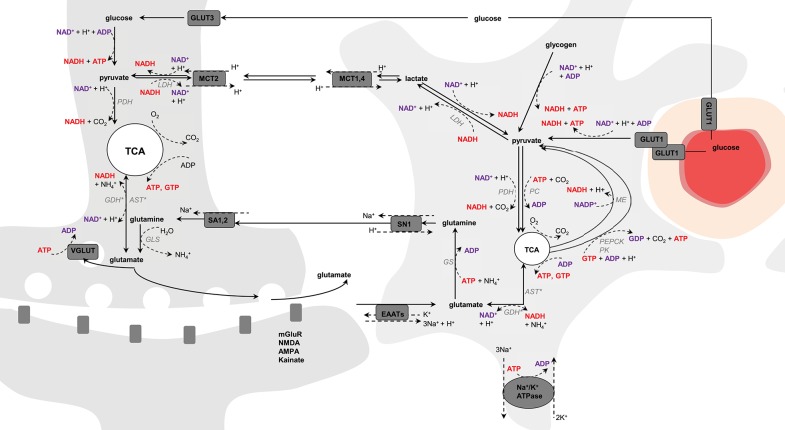
**Schematic representation of possible pathways mediating neurometabolic coupling**. Upon action potential glutamate is released in the synaptic cleft and activates the post-synaptic glutamate receptors (mGluR, NMDA, AMPA, and kainate). Glutamate molecules that are left in the synaptic cleft are transported into astrocytes via the glutamate transporters (EAAT) using the electrochemical gradient of Na^+^ (1 glutamate is transported with 3 Na^+^) and antiport of one K^+^. The Na^+^ gradient is reestablished by the Na^+^/K^+^-ATPase, an energy-dependent process (3 Na^+^ anti-transported with 2 K^+^). Glial glutamate is then converted to glutamine by glutamine synthetase (GS), an energy-dependent reaction, and is shuttled back to neurons via system N transporter (SN1) and system A transporter (SA1,2). In neuron, glutamine is hydrolyzed by glutaminase (GLS) into glutamate that is packed into vesicles by VGLUT for further glutamate release. Glutamate uptake into astrocytes is associated with glucose transport from plasma via GLUT1, and both glycolysis and oxidative metabolism via pyruvate dehydrogenase (PDH) take place. Glucose is also transported into neurons via GLUT3, where it also undergoes non-oxidative and oxidative metabolism. Produced-glutamate can re-enter the TCA cycle (i.e., glutamate oxidation) via glutamate dehydrogenase (GDH) or aspartate transaminase (AST). In astrocytes, pyruvate can be produced cataplerotically either from oxaloacetate, mediating the combined action of phosphoenolpyruvate carboxykinase (PEPCK) and pyruvate kinase (PK), or from malate by the malic enzyme (ME; i.e., pyruvate recycling). Glycolytic-derived pyruvate is converted to lactate by lactate dehydrogenase (LDH) and exchanged between neurons and astrocytes through monocarboxylate transporters (MCT1,4 and MCT2). Lactate can be converted back to pyruvate via LDH and therefore regulate the NADH/NAD^+^ redox ratio. In astrocytes, pyruvate carboxylase (PC) produces oxaloacetate from pyruvate in mediating CO_2_ fixation in an energy-dependent manner. Oxaloacetate condensates then with acetyl-CoA to produce *de novo* molecules of glutamate. The left and right cells represent an astrocyte and a neuron, respectively. The difference in TCA cycle size reflects the fact that neurons are more oxidative than astrocytes. The red circle is a blood vessel, the surrounding darker layer represents the endothelial cells and the thicker outer line is the smooth muscle cells (or pericytes). Word in bold red and bold purple corresponds to energy-producing and consuming processes, respectively. Enzymes are in gray italic. ^*^Either one or the other enzyme acts.

Once glutamate is taken by astrocytes, it can be converted to glutamine in an energy-dependent manner via the glial-specific enzyme, glutamine synthetase (GS; Derouiche and Frotscher, 1991). Glutamine is then transported to neurons via the System N transporter (SN1) in astrocytes (Chaudhry et al., 1999) and the System A transporters (SA1 and SA2) in neurons (Chaudhry et al., 2002), and converted to glutamate by glutaminase (GLS), completing therefore the glutamate-glutamine cycle (Shen et al., 1999; Zwingmann and Leibfritz, 2003), which is now accepted as a major mechanism for maintaining synaptic transmission. Therefore, while about 90% of the brain's glutamate resides in neurons (estimated between 5 and 16% in glia of the rodent brain; Tiwari et al., 2013; Lanz et al., 2014), most of the glutamine has been localized to astrocytes (Ottersen et al., 1992; Cruz and Cerdan, 1999). Glutamate can also re-enter the TCA cycle (Qu et al., 2001; Hertz et al., 2007; Sonnewald, 2014) notably by the reversible aspartate transaminase (AST) or the glial-abundant reversible enzyme glutamate dehydrogenase (GDH; Karaca et al., 2015), being oxidized for further energy or amino acid production. Consequently, the glutamate-glutamine cycle is not a stoichiometric process, as a number of amino acid molecules can be used in other metabolic pathways depending on cellular requirements (McKenna, 2007). Glutamine can diffuse out of the brain parenchyma and be used for ammonia detoxification (Zwingmann and Leibfritz, 2003). In addition, glutamate can have other fates than being converted to glutamine, such as formation of GABA and glutathione, and be synthetized from other substrates than glucose, namely lactate or ketone bodies. Amino acids can also be used for biosynthetic pathways and derived from protein degradation (McKenna, 2007; Figure 1).

## Glial support to cerebral function

### Scaffold-associated role of astrocytes

The term *astrocyte* originates etymologically from the Greek words *astron* (star) and *cyte* (cell). They belong to the general group of macroglia cells (Kettenmann and Verkhratsky, 2008), where *glia* is derived from the Greek word *gliok* (glue). Astrocytes were initially described in the middle of the nineteenth century by Rudolf Virchow (Virchow, 1856), who named *nervenkitt (nerve-putty)* the “gelatinous” substance in the brain, and later by Camillo Golgi, who hypothesized a role of astrocytes in nutrient distribution to the brain parenchyma (Golgi, 1886).

Several cytological studies on rats (Kacem et al., 1998; Mathiisen et al., 2010) and mice (Halassa et al., 2007) have shown that astrocytes are anatomically polarized cells that associate both with neurons and the vasculature: whereas perisynaptic processes contact neurons, vascular processes (or endfeet) surround intraparenchymal blood vessels (i.e., blood-brain barrier, BBB; Kacem et al., 1998). Perisynaptic processes largely express glutamate transporters (EAAT1 and EAAT2), while endfeet are more specialized in nutrient uptake and express large amounts of glucose transporters (GLUT1; Iadecola and Nedergaard, 2007). Therefore, the etymology reflects what astrocytes have traditionally been considered, satellite housekeeping cells of the brain, whose sole purpose is to serve neuronal cells in creating a favorable environment for the neurons to function efficiently. In this conventional view, astrocytes control pH and local ion homeostasis, deliver nutrients and clean neuronal waste (Nedergaard et al., 2003).

### Compartmentalization of brain energy metabolism

However, the role of astrocytes extends beyond physically supporting neurons. Interconnected via gap junctions, astrocytes form a complex functional network that detects and modulates neuronal activity, integrates and transmits surrounding signals, controls brain vasculature for nutrient delivery, and regulates and metabolizes energy substrates. Astrocytes transport glucose via GLUT1 transporters (reviewed in Maher et al., 1994). Compared to neurons, astrocytes express high levels of 6-phosphofructose-2-kinase/fructose-2,6-bisphosphatase-3 (PFKFB3), producing fructose-2,6-bisphosphate, activating in turn phosphofructokinase-1 (PFK) (Almeida et al., 2004; Herrero-Mendez et al., 2009). Astrocytes are, therefore, prone to aerobic glycolysis (Barros and Deitmer, 2010) and early studies have supported that glycolysis can be stimulated by neuronal activity (Kasischke et al., 2004), astrocytic glutamate uptake (Pellerin and Magistretti, 1994; Takahashi et al., 1995; Tsacopoulos et al., 1997) and extracellular K^+^ (Peng et al., 1994). Recently, using the genetically encoded fluorescence resonance energy transfer (FRET) lactate sensor Laconic, a lactate gradient from astrocytes to neurons was demonstrated *in vivo* (Mächler et al., 2016). The two ATPs glycolytically-produced have been proposed to serve the energetic demands associated with glutamatergic activity in fueling both the Na^+^/K^+^-ATPase pump coupled to glutamate transport (consuming one ATP) and the glutamine synthetase (consuming another ATP) for the maintenance of the glutamate-glutamine cycle (Pellerin and Magistretti, 1994). On the other hand, neurons constantly degrade PFKFB3 (Almeida et al., 2004; and reviewed in Bolaños et al., 2010) and neuronal activation of PFKFB3 was shown to lead to oxidative stress and neuronal apoptosis (Herrero-Mendez et al., 2009). Astrocytic-produced lactate was therefore proposed to alternatively fuel neurons for oxidative metabolism during neuronal activity, in line with stimulation of glycolysis by K^+^ (Peng et al., 1994; Bittner et al., 2011), by glutamate (Pellerin and Magistretti, 1994), and increased lactate efflux by K^+^ (Sotelo-Hitschfeld et al., 2015) and NH^+4^ (Provent et al., 2007; Lerchundi et al., 2015), while neuronal glucose to be diverted into the pentose phosphate pathway for antioxidant defense during enhanced work at the respiratory chain (Bouzier-Sore and Bolaños, 2015). The study by Sibson et al. in the rat brain suggesting 1:1 stoichiometry between neuronal glucose oxidation and the glutamate-glutamine cycle rate (Sibson et al., 1998) provided support to this early view of compartmentation of brain metabolism. However, this hypothesis did not consider (or exclude) stimulation of glial oxidative metabolism to support increased neurotransmission rates. Yet, hexokinase was shown to be highly expressed in neurons as compared to astrocytes (Lundgaard et al., 2015), which is consistent with the ability of neurons to rapidly upregulate their glycolytic activity to fuel energy demand. In line with this, it was reported that increased glycolysis occurs for example in nerve terminals of bicuculline-treated rats (Patel et al., 2014), and in cultured neurons exposed to high K^+^ (Peng and Hertz, 2002).

### Stimulation of glial oxidative metabolism *in vitro*

Although glycolytic activity is higher in glia than in neurons, astrocytes express an important number of enzymes involved in the TCA cycle, suggesting substantial oxidative capacity. In particular, about 45 and 16% of gene expression in astrocytes is dedicated to energy homeostasis and energy substrate transport, respectively, and TCA cycle related genes (i.e., citrate synthase, aconitase, isocitrate dehydrogenase, oxoglutarate dehydrogenase, dihydrolipoamide s-succinyltransferase, succinyl-CoA ligase, succinate dehydrogenase, fumarase, malate dehydrogenase) have larger expression in astrocytes than in neurons (Lovatt et al., 2007). Moreover, an important amount of small mitochondria was detected in the fine astrocytic processes (Derouiche et al., 2015) and shown to co-localize with EAAT (Genda et al., 2011; Jackson et al., 2014), which might facilitate coupling of astrocytic respiration with glutamate uptake (Eriksson et al., 1995). Additionally, elevation in astrocytic Ca^2+^ coincide with mitochondria position within the processes (Jackson and Robinson, 2015), and a rise in mitochondrial Ca^2+^ concentration might stimulate TCA cycle activity and thus ATP production (Wan et al., 1989; Denton, 2009). Consistent with glial metabolic activation by neuronal activity (Eriksson et al., 1995) and stimulation of Na^+^/K^+^-ATPase by extracellular K^+^ (Hajek et al., 1996; Honegger and Pardo, 1999), the fraction of glutamate that is metabolized through glial TCA cycle was reported to increase with extracellular glutamate application to astrocytes (McKenna et al., 1996).

### Glial oxidative metabolism *in vivo*

A few *in vivo* studies have investigated the metabolic involvement of glia during neuronal activation. In particular, autoradiography studies using the glial-specific energy substrate acetate demonstrated increased glial oxidative metabolism during both acoustic (Cruz et al., 2005) and photic (Dienel et al., 2007b) stimulations in awake rats. Similarly, positron emission tomography (PET) along with [1-^11^C]acetate infusion showed increased astrocytic oxidative metabolism during infraorbital nerve stimulation of anesthetized rats and visual stimulation in humans (Wyss et al., 2009). Using dual photon fluorescence confocal microscopy, Lind et al. showed that during trigeminal nerve stimulation of anesthetized mice about 70% of astrocytes respond to stimulation with Ca^2+^ increase (Lind et al., 2013). With the same methodology the glial activation map was reported to resemble that of neurons during rat paw stimulation, suggesting both neuronal, and glial topographical representation of the body in the cortex (Ghosh et al., 2013), and pointing to significant glial activation during neuronal activity.

While these techniques present high spatial resolution, they are associated with some disadvantages, such as the need of radioactive tracers and ionizing radiations (PET, autoradiography), invasiveness (dual photon fluorescence confocal microscopy, FRET, autoradiography), potential cellular toxicity (FRET), the lack of absolute quantification in the case of FRET (as signal magnitude directly rely on the number of molecules of interest binding the sensor), and the lack of chemical specificity (PET, autoradiography) for providing quantitative information on metabolic pathways underlying oxidative metabolism. Currently, the development of tracers detectable by MRS, such as ^13^C-labeled substrates, the improvement of detection modalities (Henry et al., 2003), the progress in building adequate mathematical models (Gruetter et al., 2001) along with the increase in magnetic field strength render possible detailed compartmentalized metabolic flux characterization *in vivo* in a non-invasive manner and with minimal assumptions (Duarte et al., 2011; Duarte and Gruetter, 2013; Dehghani et al., 2016; Sonnay et al., 2016, 2017). In particular direct detection of ^13^C-labeled compounds (^13^C MRS) provides quantitative assessment of major metabolic pathways including glycolysis, TCA cycle, glutamate-glutamine cycle and pyruvate carboxylase (Gruetter et al., 2001; Henry et al., 2006; Duarte et al., 2011). Direct detection of ^13^C-labeled compounds is a technique that has been robustly implemented and can be applied to small animal metabolism studies in the whole brain (Duarte et al., 2011) or in specific cerebral regions (Patel et al., 2005a; Sonnay et al., 2016, 2017).

## Dynamic ^13^C magnetic resonance spectroscopy (^13^C MRS)

Nuclear magnetic resonance (NMR) is a non-ionizing and non-invasive technique based on the magnetic properties of spin-containing nuclei. This methodology can be used in both clinical settings (e.g., Prichard et al., 1991; Rothman et al., 1992; Gruetter et al., 1994, 1998a, 2001; Shen et al., 1999; Gruetter, 2002; Lebon et al., 2002; de Graaf et al., 2004; Mangia et al., 2007; Oz et al., 2007, 2015; Lin et al., 2012; Schaller et al., 2013, 2014; Bednařík et al., 2015) and pre-clinical studies (e.g., Mason et al., 1992; Hyder et al., 1996, 1997; Sibson et al., 1998, 2001; Choi et al., 2002; Henry et al., 2002; Patel et al., 2004, 2005a; Deelchand et al., 2009; Duarte and Gruetter, 2013; Duarte et al., 2011, 2015; Mishkovsky et al., 2012; Just et al., 2013; Bastiaansen et al., 2013, 2015; Lanz et al., 2014; Sonnay et al., 2015, 2016, 2017). However, in most animal applications it requires anesthesia, which can modify the coupling between neuronal activity, brain metabolism and vascular regulation of blood flow (Masamoto and Kanno, 2012; Sonnay et al., 2017, and references therein).

Several nuclei can be used to investigate brain metabolism, notably ^1^H, ^31^P, or ^13^C. In the case of phosphorus, ^31^P MRS is used to investigate energy metabolism by providing information on the energy status of endogenous phosphate compounds, namely ATP, ADP, and PCr (e.g., Zhu et al., 2009). ^1^H MRS is based on local environment-dependent ^1^H present in metabolites (discarding water that is several orders of magnitude more concentrated) and assesses changes in total metabolite concentrations (generally in the mM range) involved in energy metabolism, osmoregulation, membrane metabolism and myelination (reviewed in Duarte et al., 2012). The main challenge associated with ^1^H MRS is the complexity of the neurochemical profile composed of several overlapping metabolite signals on a relatively small frequency range (i.e., 4–5 ppm) that is to be analyzed (de Graaf, 1998). An extension of this technique is ^1^H functional MRS (fMRS) that focuses on time-dependent changes in metabolite concentrations, which are associated with metabolic pathways during brain activity (Prichard et al., 1991; Mangia et al., 2007; Lin et al., 2012; Just et al., 2013; Schaller et al., 2013, 2014; Bednařík et al., 2015). The difficulty associated with the detection of small concentration changes occuring during neuronal activation can yet be overcome by using high magnetic field MR system (≥9.4 T) to increase spectral resolution and sensitivity.

Direct ^13^C MRS can measure ^13^C isotope incorporation (or fractional enrichment, FE) over time into different molecules and into specific positions within the same molecule (i.e., ^13^C isotopomers), with signals distributed over a large chemical shift range, namely about 200 ppm. Since the ^13^C isotope has a natural low abundance (1.1%), a low background signal is detected. However, as ^13^C gyromagnetic ratio is ¼ of that of ^1^H, ^13^C MRS is an insensitive technique as compared with ^1^H MRS. This is the reason why ^13^C tracers are infused in a substantial amount to enable proper signal detection. The ^13^C MRS detection threshold *in vivo* is typically in the mM range and it is usually not possible to measure TCA cycle intermediates that are present in smaller quantities. However, amino acids (e.g., glutamate, glutamine, and aspartate), which are in exchange with TCA cycle intermediates, are present at higher concentrations and can, therefore, be measured and used for metabolic modeling and thus for estimation of fluxes across major biochemical pathways.

On the other hand, dynamic nuclear polarization (DNP) can be used to increase ^13^C polarization of ^13^C-labeled substrates, and thus offers potentially tremendous signal enhancement and detection of ^13^C labeling in tissue's TCA cycle intermediates, such as 2-oxoglutarate in the brain (Mishkovsky et al., 2012), or citrate in the heart (Schroeder et al., 2009; Bastiaansen et al., 2015). While such a technique can probe metabolism *in vivo* with high sensitivity and a time resolution of 1 s, the acquisition window is limited to approximately a minute (Comment, 2016), since MR acquisition needs to be performed within the time decay (seconds) of the enhanced nuclear polarization, and detection of downstream compounds depends notably on the turnover rates and on the concentration of labeled metabolites produced within the recording period (discussed in Mishkovsky et al., 2012). Moreover, further developments are required to actually translate the detection and measurement of hyperpolarized ^13^C *in vivo* to quantification of metabolic fluxes (Bastiaansen et al., 2013).

The strong heteronuclear scalar coupling between ^13^C and ^1^H nuclei complicates the spectra (i.e., splitting of ^13^C resonances in multiplets with reduced peak height) and reduces the sensitivity, so that additional hardware is required for ^1^H decoupling during acquisition (discussed in Henry et al., 2006). Homonuclear ^13^C couplings are also observable and the presence of ^13^C multiplets depends on the simultaneous presence of ^13^C spins in the same molecule at adjacent positions. Although assessment of ^13^C multiplets with high temporal resolution is challenging because of low signal amplitude, their inclusion in mathematical modeling (i.e., bonded cumomer approach) improves reliability and independency of the estimated brain metabolic fluxes (Shestov et al., 2012; Tiret et al., 2015; Dehghani et al., 2016).

The evaluation of ^13^C enrichment curves over time of carbon containing molecules requires the use of multi-compartment models describing best the data (de Graaf et al., 2003, 2004; Patel et al., 2004, 2005a, 2010; Henry et al., 2006; Lanz et al., 2013). As a simplified view of the real biochemical network, a model is a set of metabolite pools interconnected by the major biochemical reactions that are associated with metabolic fluxes. Each pool is associated with certain labeling positions in the atomic chain of one metabolite synthesized downstream from the infused compound: therefore there are at least as many labeling equations as carbon positions. The model contains the measurable entities and the non-measurable pools that are present in lower concentrations (e.g., TCA cycle intermediates) and that reach steady-state labeling much faster than larger pools, such as amino acids (Uffmann and Gruetter, 2007). The model can combine isoenzymes, parallel pathways that result in the same labeled pools, intermediate pools that equilibrate rapidly (e.g., pyruvate/lactate) or which enzymes are assumed not to be involved in other processes (e.g., glycolysis). According to the complexity to reach, the model can assess reversibility of reactions, sub-compartmentation or transport across membranes (reviewed in Henry et al., 2006). Based on the known biochemical reactions, a model should, therefore, be as simple as possible and as complex as necessary to describe measured parameters, focusing on the relevant pathways leading to metabolic observations and neglecting the influence of others (e.g., presence of cofactors, much slower reactions).

Derivation of metabolic fluxes is usually performed using dynamic positional enrichment (total amount of label accumulated at individual position without distinguishing within multiplets; de Graaf et al., 2004; Duarte et al., 2011; Sonnay et al., 2016, 2017). The metabolic model describing ^13^C labeling is solved mathematically by a set of coupled linear differential mass-balance equations describing the system at equilibrium (i.e., metabolic but not isotopic steady-state). It assumes mass and energy conservation (i.e., constant fluxes and pool size over time), instant and uniformly labeling of the pools, and equal probability for a labeled or non-labeled molecule to enter and leave a pool (Mason et al., 1992 and see Lanz et al., 2013 for mathematical details). This latter assumption is plausible given that the biochemical reactions are fast compared to the temporal resolution of the MR acquisition techniques. Note that the metabolic steady-state assumption might not necessary hold under certain conditions (Lanz et al., 2014).

### Isotopomers from [1,6-^13^C_2_] glucose

**Figure 2:**
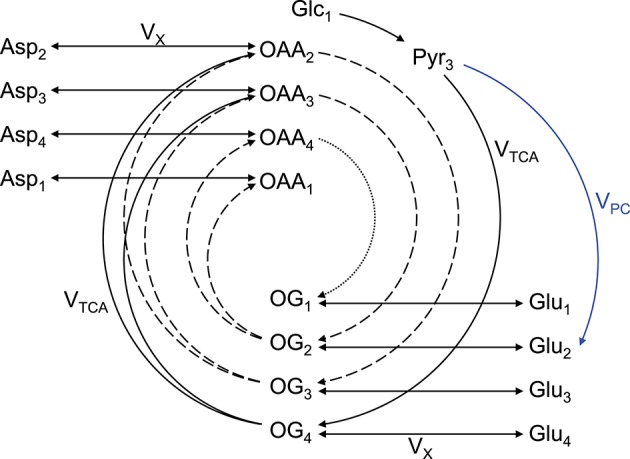
**Schematic overview of labeling transfer during [1,6-^13^C_2_]glucose or [1-^13^C]glucose infusion**. The splitting of the labeling after OG is due to the symmetry at the succinate and fumarate level. The indexes represent the carbon positions that become labeled. The first, second, and third turns of the TCA cycle are represented by solid, dashed, and dotted lines, respectively. The glial specific reaction driven by pyruvate carboxylase (V_PC_) labels position C2 and dilutes position C3. Glc, glucose; Lac, lactate; Pyr, pyruvate; Asp, aspartate; OAA, oxaloacetate; OG, 2-oxoglutarate; Glu, glutamate; Gln, glutamine; V_TCA_, TCA cycle rate; V_X_, transmitochondrial exchange rate (modified from Lanz et al., 2013).

An extensively used substrate for two-compartment (i.e., neurons vs. astrocytes) brain metabolism investigation is [1,6-^13^C_2_]glucose (Lanz et al., 2013). On a general principle, [1,6-^13^C_2_]glucose crosses the BBB through facilitate glucose transporters (GLUT), is taken up by neurons and astrocytes, and 1 mole of glucose is metabolized through glycolysis (cytosolic reaction) to produce 2 moles of [3-^13^C]pyruvate (1 mole of [1-^13^C]glucose only produces 1 mole of [3-^13^C]pyruvate, resulting in two-fold reduced ^13^C labeling of pyruvate). Two moles of [2-^13^C]acetyl-CoA is then synthetized by PDH. Then in the first turn of the mitochondrial TCA cycle, [2-^13^C]acetyl-CoA will label position C4 of 2-oxoglutarate. Due to the transmitochondrial exchange mediated notably by GDH and AST, cytosolic glutamate C4 is in turn labeled (the carbon positions are maintained). Due to the symmetry of the succinate and fumarate molecules, half of the labeling in position C4 of 2-oxoglutarate is transferred with equal probability to positions C2 and C3 of oxaloacetate, leading to the formation of [2-^13^C]aspartate and [3-^13^C]aspartate from oxaloacetate by AST. In the second turn of the TCA cycle, labeled molecules of oxaloacetate combine again with (labeled or unlabeled) molecules of acetyl-CoA, resulting in the formation of [2-^13^C]glutamate and [3-^13^C]glutamate. Then in the third turn of the TCA cycle, half of the carbons of position C3 of 2-oxoglutarate are transferred to position C2, while the other half stays at position C3. Meanwhile carbons at position C2 are transferred to position C1 of 2-oxoglutarate or lost as CO_2_. The aspartate pool is enriched at position C1, C2, C3 and C4. In astrocytes, [3-^13^C]oxaloacetate can be formed from [3-^13^C]pyruvate by PC, leading to the formation of [2-^13^C]2-oxoglutarate and [2-^13^C]glutamate. Therefore, [1,6-^13^C_2_]glucose, as [1-^13^C]glucose, allows differentiating astrocytes and neurons in labeling positions C2 and diluting positions C3 (with ^12^C) of glutamate via the glial specific enzyme PC, but enhances signal detection at a given resonance by two-fold compared to [1-^13^C]glucose (two times more [3-^13^C]pyruvate at the end of glycolysis). In the glutamate-glutamine cycle, the carbon positions are maintained. As the labeling persists in the TCA cycle, multiple carbon positions become labeled (Figure 2).

Other labeled substrates can be used to study brain metabolism by direct ^13^C MRS *in vivo* depending on the labeling pattern. For instance, [2-^13^C]glucose in the human brain allowed labeling the carboxyl groups of glutamate and glutamine (i.e., positions C1 and C5), and aspartate (i.e., positions C1 and C4; Li et al., 2016). However, half of labeling is rapidly lost as CO_2_. Using uniformly labeled glucose ([U-^13^C]glucose) increases the splitting of the resonance C4 of glutamate and glutamine due to J-coupling with position C5 (Henry et al., 2003). [3-^13^C]lactate can be used as an alternative to [1,6-^13^C_2_]glucose, but results in a reduced amount of labeling molecules because of a lower lactate entry into the brain as compared to [1,6-^13^C_2_]glucose (Duarte et al., 2015). [2-^13^C]acetate has the particularity of being metabolized almost exclusively in glia. It produces [2-^13^C]acetyl-CoA and the large neuronal glutamate pool becomes labeled via glial [4-^13^C]glutamine. Therefore, [2-^13^C]acetate allows increasing the sensitivity of the measurements of glial oxidative metabolism and the glutamate-glutamine cycle in minimizing the bias toward the large neuronal glutamate pool (Patel et al., 2010; Lanz et al., 2014). To obtain more precise measurements on neuronal and glial oxidative metabolism, [1,6-^13^C_2_]glucose can be co-infused with [1,2-^13^C_2_]acetate. However, advanced modeling approaches are needed to account for the additional homonuclear coupling brought by this double infusion (Deelchand et al., 2009).

### Compartmental models of brain metabolism

#### One-compartment model

**Figure 3:**
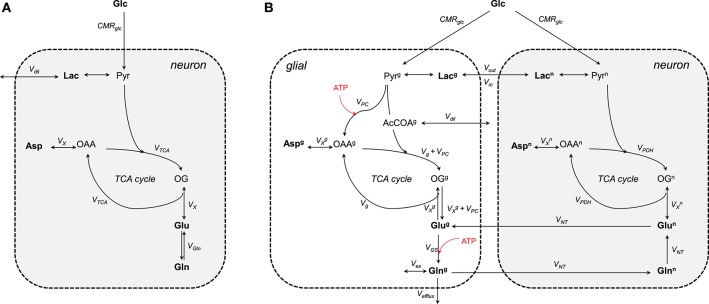
**Schematic view of the (A)** one- and **(B)** two-compartments model. **(A)** As glutamate is mainly located in neurons, the one-compartment model represents neurons. This model is characterized by the total TCA cycle rate (V_TCA_), the transmitochondrial exchange rate (V_X_) and to some extent the neurotransmission rate modeled by the glutamine exchange rate (V_Gln_). A dilution factors (V_dil_) regulates the amount of lactate entering and leaving the cell. **(B)** The two compartments are linked by the glutamate-glutamine cycle. Plasma glucose is transported into the cells down its concentration gradient via GLUT1 (in glial) and GLUT3 (in neurons) transporters. Once inside glucose is processed through glycolysis into pyruvate. Pyruvate enters then the TCA cycle to produced energy and amino acids. The glial-specific enzyme, pyruvate carboxylase (PC), produces *de novo* molecules of glutamate by carboxylation of pyruvate into oxaloacetate. Glc, glucose; Lac, lactate; Pyr, pyruvate; Asp, aspartate; OAA, oxaloacetate; OG, 2-oxoglutarate; Glu, glutamate; Gln, glutamine; CMR_glc_, cerebral metabolic rate of glucose consumption; V_PDH_, neuronal TCA cycle rate; V_g_, glial full oxidation of pyruvate; V_PC_, pyruvate carboxylase; V_GS_, glutamine synthetase; V_NT_, neurotransmission rate; V_X_, transmitochondrial exchange rate; V_ex_, exchange rate with glial glutamine; V_efflux_, loss of glial glutamine (or ammonia detoxification = V_PC_); V_dil_, in **(A)** dilution at the level of lactate and in **(B)** dilution rate from glial-specific substrates. Boldface indicates MR-measurable metabolites. The superscripts n and g indicate neuron and glial, respectively. ATP-dependent reactions are indicated with the red arrows.

The one-compartment model (Figure 3A) was the first metabolic model used to describe the turnover curves following infusion of [1-^13^C]glucose along with ^1^H-[^13^C] MRS (i.e., detection of ^1^H attached to ^13^C; Mason et al., 1992; Rothman et al., 1992; Hyder et al., 1996). As most of the glutamate is located in neurons (Ottersen et al., 1992), this model is assumed to mainly represent neuronal TCA cycle activity. It allows assessing the TCA cycle rate (V_TCA_), the trans-mitochondrial flux (V_X_) and a dilution flux at the level of lactate (V_dil_, accounting for the utilization of unlabeled substrates; Mason et al., 1992) from fitting glutamate and glutamine C4, and Glx (glutamate+glutamine) C3. Although the astrocytic compartment is not represented in this model, the neurotransmission rate can be modeled with a glutamine exchange rate (V_Gln_; Henry et al., 2002). While ^1^H-[^13^C] MRS studies provided an initial measure of local oxidative metabolism, they were limited to the number of detected ^13^C isotopomers and were dependent on several assumptions regarding glial fluxes (Patel et al., 2004; de Graaf et al., 2004). With the rapid improvement of MRS methodology and sensitivity (Gruetter et al., 1994, 1998a; Henry et al., 2003, 2006), detection of carbon position C2, C3, and C4 (and their splitting) of glutamate and glutamine became possible (i.e., direct detection of ^13^C-labeled compounds) resulting to the development of two-compartments models with minimum assumptions regarding glial fluxes (Gruetter et al., 2001).

#### Two-compartment models

Two-compartment models describe compartmentalization of brain energy metabolism between glutamatergic neurons and astrocytes linked by the glutamate-glutamine cycle (Figure 3B). In contrast to the one-compartment model, this model can estimate up to nine independent fluxes from the labeling curves of glutamate, glutamine, and aspartate (Duarte et al., 2011; Sonnay et al., 2016, 2017) resulting in up to thirteen parameters that can be assessed when combining some of the fitted fluxes. More precisely, the fitted parameters are the neurotransmission rate (V_NT_; i.e., glutamate-glutamine cycle representing neurotransmission), the neuronal and glial TCA cycle (i.e., V_PDH_ and V_g_, representing glial full pyruvate oxidation), pyruvate carboxylase activity (V_PC_), the trans-mitochondrial exchange rates describing the combined effects of AST, GDH and transport across mitochondrial membrane occurring in neurons (${\text{V}}_{\text{X}}^{\text{n}}$) and in glia (${\text{V}}_{\text{X}}^{\text{g}}$), glial glutamine exchange (V_ex_), pyruvate/lactate influx from plasma (V_in_) and dilution from glial specific substrates (i.e., acetate and fatty acids) (V_dil_). In addition, calculated fluxes are total glial TCA cycle activity (${\text{V}}_{\text{TCA}}^{\text{g}}$ = V_g_ + V_PC_), the glutamine synthetase rate (V_GS_ = V_NT_ + V_PC_), the total cerebral metabolic rate of glucose oxidation (CMR_glc(ox)_ = [${\text{V}}_{\text{TCA}}^{\text{n}}\;+\;$${\text{V}}_{\text{TCA}}^{\text{g}}\;+\;$V_PC_]/2) and the pyruvate/lactate out-flux from the brain parenchyma (V_out_ = 2CMR_glc_-2CMR_glc(ox)_ + V_in_). CMR_glc_ is the cerebral metabolic rate of glucose and is usually determined together with the apparent maximum transport (T_max_) and the apparent Michaelis constant of glucose transport K_t_ using labeling of plasma and brain glucose (Duarte et al., 2011; Sonnay et al., 2016, 2017). In glial cells, GLS is neglected because the net ^13^C labeling follows the direction of glutamine synthesis. V_ex_ can exchange with unlabeled glutamine of undefined origin (Oz et al., 2004) or the proposed ^1^H MR invisible but ^13^C labeled glutamine pool (Duarte and Gruetter, 2013). Besides the contribution to amino acid synthesis, V_PC_ also represents glutamine efflux (V_efflux_) from the brain (i.e., ammonia disposal) and maintains the mass balance in the glial TCA cycle (Lee et al., 1998). Glutamate oxidation in the model is possible through V_X_ (composite representation of AST and GDH). Note that both V_Gln_ and V_NT_ represent the glutamate-glutamine cycle. One is rather used in the one-compartment model (V_Gln_) as an exchange rate between glutamate and glutamine, while the other in the two-compartment model (V_NT_).

#### Flux information brought by the turnover curves of amino acids

**Figure 4:**
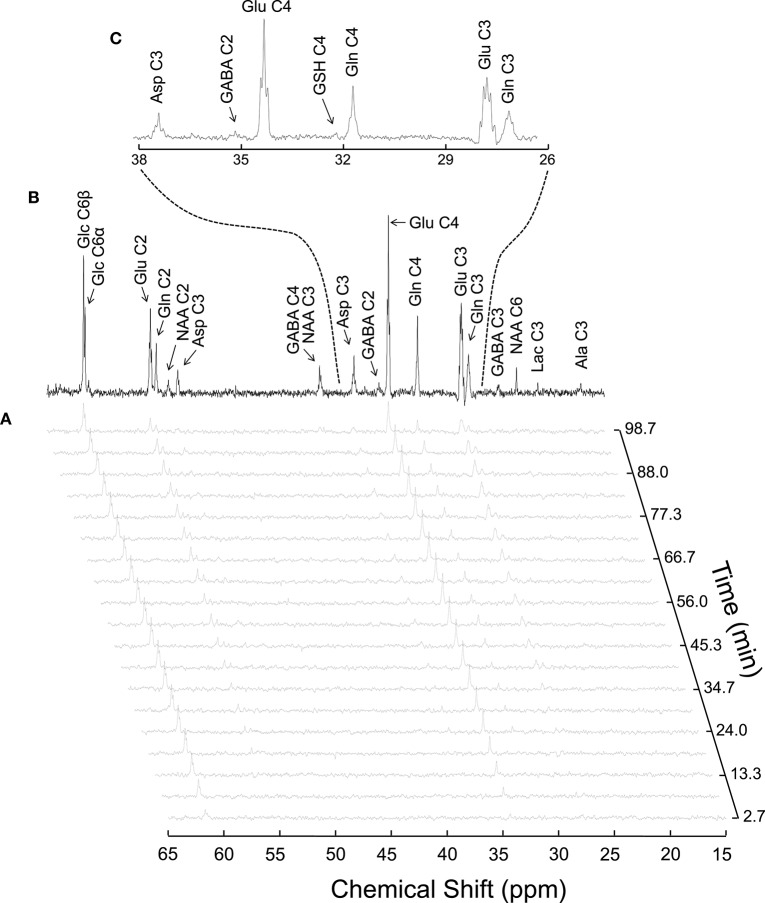
**Typical ^13^C MRS spectra acquired *in vivo* in the rat brain during [1,6-^13^C_2_]glucose infusion at 14.1T (reproduced from Duarte et al., 2011)**. Panel **(A)** shows a time course of ^13^C labeling with a temporal resolution of 5.3 min. The spectrum in **(B)** was acquired for 1.8 h, starting 3.5 h after the infusion onset. Panel **(C)** is an expansion of B depicting multiplets originated from isotopomers of glutamine (Gln), glutamate (Glu) and aspartate (Asp).

Labeling of a particular nucleus depends on pool size and the numbers of upstream and downstream fluxes (Henry et al., 2006), affecting therefore the shapes of the turnover curves differently. Glutamate is the most concentrated amino acid observed by ^13^C MRS (Figure 4). Glutamate labeling depends on both V_TCA_ and V_X_, meaning that these two fluxes play an important role in defining glutamate turnover. While glutamate C4 relies on the composite flux V_gt_, that is V_X_.V_TCA_/(V_X_ + V_TCA)_, glutamate C3 and C2 depend on independent contribution of V_gt_, V_X_, and V_TCA_ (reviewed in Lanz et al., 2013), because they are labeled in the second turn of the TCA cycle. Therefore, the information concerning V_X_ is mainly stored in the initial slopes of the turnover curves of position C4, C3, and C2 of glutamate (for ${\text{V}}_{\text{X}}^{\text{n}}$) and glutamine (for ${\text{V}}_{\text{X}}^{\text{g}}$). In the special case of ${\text{V}}_{\text{X}}^{\text{g}}$, this flux is particularly difficult to estimate, because labeled molecules from the glial TCA cycle into glial glutamate can also be transferred via V_PC_ (Figure 2). The value of V_X_ has been matter of debate for a long time, as it has been considered by some to be much larger than V_TCA_ (Mason et al., 1992; Patel et al., 2004; Yang et al., 2009), but it was also estimated to be on the same order of magnitude of V_TCA_, as reflected in a delay in the C3 and C2 labeling relative to C4 (Gruetter et al., 2001; Oz et al., 2004; Duarte et al., 2011; Duarte and Gruetter, 2013; Lanz et al., 2014; Sonnay et al., 2016).

The neurotransmission rate, V_NT_, represents the conversion of glutamate to glutamine and vice versa. In this process the carbon positions are maintained. Therefore, V_NT_ mostly depends on the relative steady-state enrichment of the turnover curves of glutamate and glutamine: the closer they are to each other, the faster V_NT_.

The first turn of the TCA cycle results in label transfer from glucose to glutamate (via V_X_). Usually glutamate C4 is the first detectable peak in a spectrum during an experiment, as it appears within the first 5 min of infusion (Patel et al., 2005a; Duarte et al., 2011; Sonnay et al., 2016 and reviewed in de Graaf et al., 2003). Then, in the subsequent TCA cycle label is transferred from position C4 to C3 and C2. In neurons, V_PDH_ will therefore rely on the slope and the steady-state enrichment of the turnover curves of position C4, C3, and C2 of glutamate and glutamine, as labeling from glutamate is transferred to glutamine. As aspartate is mainly labeled via transamination of the TCA cycle intermediate oxaloacetate, the slope and the steady-state enrichment of position C3 and C2 of aspartate are further affected by V_PDH_. The slopes of the labeling curves reflect, the rate of V_PDH_. In glia the situation is different, since label dilution due to V_PC_ can occur. V_PC_ dilutes position C3 and labels position C2 of glutamate and glutamine. Therefore, fast V_g_ results in a relatively high and steep glutamine C3 turnover curve (V_g_ has to be fast to counterbalance the loss due to V_PC_).

Diluting position C3 and labeling position C2 of glutamate and glutamine, the measurement of V_PC_ relies on the relative curves of position C3 and C2 of glutamate and glutamine, and the assumption that glutamate is mainly neuronal and glutamine mainly glial. High C3 and low C2 labeling is associated with slow V_PC_, while high C2 and low C3 labeling reflect rather increased V_PC_. Therefore, when the FE of glutamine C2 approaches that of C4, either PC activity is high compared to PDH, and/or glial-specific dilution of acetyl-CoA (V_dil_) occurs.

As mentioned above, V_dil_ reflects dilution of the acetyl-CoA pool with specific unlabeled glial substrates. Notably, V_dil_ dilutes glial acetyl-CoA ^13^C labeling relative to its precursor pyruvate. As the position C4 of glutamate and glutamine only receives labeling from acetyl-CoA, dilution at this point would lead to a lower (steady-state) C4 labeling. Since glutamate and glutamine are mainly present in neurons and glia, respectively, V_dil_ is also responsible for lower FE of position C4, C3, and C2 of glutamine compared to glutamate. However, the effect of V_dil_ on enhancing the labeling difference between glutamate and glutamine is counteracted by V_NT_, which represents the glutamate-glutamine cycle. The faster the rate of V_NT_, the more similar will be the labeling of glutamate and glutamine. Note that V_dil_ in glial acetyl-CoA can result in glutamine C2 being similar or larger than glutamine C4, which has been observed in some studies (discussed in Duarte et al., 2011).

V_ex_ represents an exchange between two putative glutamine pools, one of which is not released to neurons and may account for a continuous slow increase in FE over time (Duarte and Gruetter, 2013). V_ex_ can be in exchange with a ^1^H MR invisible but ^13^C labeled glutamine pool (Hancu and Port, 2011) or with unlabeled amino acids from the blood (i.e., glutamine), as astrocytes envelop capillaries (Oz et al., 2004). This second glutamine pool could be associated with biosynthetic pathways, which have rates much slower than mitochondrial energy metabolism (McKenna, 2007). The effect of V_ex_ is in practice observable near the end of an experiment, when the labeling of glutamine still increases, while glutamate is at steady-state.

The difference V_out_-V_in_ directly reflects whether the labeling from plasma glucose is enough to fuel the whole downstream metabolism since V_out_-V_in_ = 2CMR_glc_-2CMR_glc(ox)_. Therefore, if mitochondrial metabolism is faster than glycolysis, oxidation of additional substrates, such as lactate, must occur under certain conditions (Sonnay et al., 2017). In resting human brain, however, the brain exports lactate to the blood stream (discussed in Dienel, 2012).

The above descriptions are purely indicative of what happens for each flux independently. Experimental data is a linear combination of many fluxes, which will adjust during fitting process to best describe the turnover curves.

## Increased glial and neuronal glucose oxidation with neuronal activity assessed by ^13^C MRS

The first ^13^C MRS data acquired *in vivo* upon stimulus-induced brain activity were modeled using a one-compartment model and reported a marked increase in total TCA cycle activity in the somatosensory cortex of stimulated rats compared to rest (Hyder et al., 1996, 1997). The following experiment consisted on measuring neuronal CMR_glc(ox)_ under three different anesthesia-induced activity states, namely pentobarbital (deep), α-chloralose (moderate) and morphine (light) (Sibson et al., 1998). In this study, energy metabolism was found to be coupled to the rate of the glutamate-glutamine cycle (representing glutamatergic neurotransmission, V_NT_ in Figure 3) in a proportion of 1:1 above isoelectricity, and the main assumption of the model was driven by the astrocyte-neuron lactate shuttle (ANLS) hypothesis according to which two glycolytic ATP are rapidly produced to fuel both glutamate uptake via the Na^+^/K^+^-ATPase and glutamine synthetase (Pellerin and Magistretti, 1994). According to this model, no stimulation of oxidative metabolism should occur in glia, in contrast to neurons. Later several studies in rat brain (Patel et al., 2004, 2005a; de Graaf et al., 2004), assuming V_PC_ as a fixed fraction of V_GS_ (Sibson et al., 2001) and ${\text{V}}_{\text{TCA}}^{\text{g}}$ as a fraction of total V_TCA_ (van den Berg and Garfinkelm, 1971; Lebon et al., 2002), corroborated the stoichiometry. However, it should be noted that constraining the value of V_PC_ to V_GS_ and ${\text{V}}_{\text{TCA}}^{\text{g}}$ to the total V_TCA_ implies an effective coupling between glial oxidative metabolism and neuronal function.

Indeed, the astrocytic processes engulfing synapses are capable of sensing increased synaptic activity (Iadecola and Nedergaard, 2007; Cheung et al., 2014) and to stimulate metabolism in local mitochondria (Eriksson et al., 1995; Jackson et al., 2014). The ^13^C MRS study by Gruetter et al. (2001) in the human brain, modeling for the first time the occurrence of glucose oxidation in the glial compartment, indeed demonstrated that a significant fraction (≈21%) of glucose is also oxidized in astrocytes (Gruetter et al., 2001). Using a similar model, glial oxidation and pyruvate carboxylase activity was shown to significantly contribute also to total glucose oxidation in awake animals (Oz et al., 2004), and rats anesthetized with α-chloralose (Duarte et al., 2011), pentobarbital (Choi et al., 2002) and thiopental (Sonnay et al., 2017). In these rodent studies, going from the awake state to deep anesthesia, glial metabolism was found to account for 30–40% of total oxidative metabolism.

**Figure 5:**
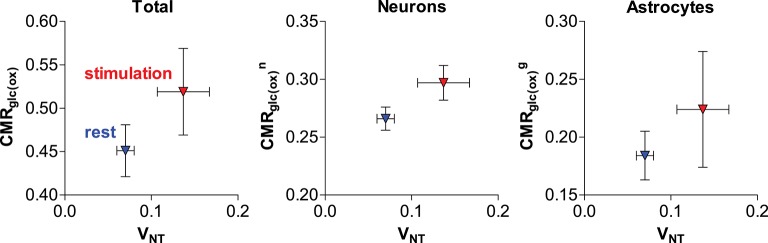
**Relation of estimated total, neuronal and glial glucose oxidative metabolism to the glutamate-glutamine cycle in the rat cortex anesthetized with α-chloralose (originally reported in Sonnay et al., 2016)**. Metabolic fluxes are in μmol/g/min. Average fluxes across the resting (in blue) and stimulated (in red) group are shown with associated SD.

Recently, our group further addressed the issue of glial and neuronal oxidative metabolism coupled to neuronal activity. In particular, we first measured the cortical changes in metabolic fluxes induced by electrical stimulation of the four paws of rats. We observed a similar increase (in absolute terms) of both glial and neuronal oxidative metabolism resulting from the increase in glutamate-glutamine cycle rate (Figure 5; Sonnay et al., 2016). Moreover, about 37% of total glucose oxidation, i.e., CMR_glc(ox)_, occurred in astrocytes at rest, 39% during stimulation and ΔCMR_glc(ox)_/ΔV_NT_ ≈ 1. Interestingly in this study, as well as in Patel et al. (2005b), PC did not vary with V_NT_, suggesting that *de novo* synthesis of amino acids is not required for increases of the glutamate-glutamine cycle, neither there is increase in glutamine loss from the cortical tissue. Indeed Patel and Tilghman reported that glutamate can stimulate pyruvate carboxylation (Patel and Tilghman, 1973). Instead, glutamate could be oxidized in astrocytes to compensate for the high cost of glutamate uptake during neurotransmission (McKenna, 2013).

In the study by Sonnay et al. (2016) the resulting incremental ATP produced by glucose oxidation was in excess of the increase in ATP required by the glutamate-glutamine cycle *per se* (i.e., glial Na^+^/K^+^-ATPase extrusion of Na^+^ that is co-transported with glutamate and glial glutamine synthetase activity). While the fate of neuronal ATP is likely involved in supporting other functions than the glutamate-glutamine cycle, such as stabilization of membrane potentials and restoration of ion (Na^+^, K^+^, and Ca^2+^) gradients across the cell membrane (Attwell and Laughlin, 2001), the role of the considerable amount of ATP produced in glial cells (as estimated from recent ^13^C MRS experiments in rodents; Duarte et al., 2011, 2015; Sonnay et al., 2016, 2017) is still unclear and likely extends beyond fueling glutamine synthetase, the Na^+^/K^+^-ATPase and the Ca^2+^-ATPase (Fresu et al., 1999). K^+^ uptake has been recently suggested to fully account for astrocytic energy consumption (DiNuzzo et al., 2017). However, the simulations by DiNuzzo et al. are still unable to account for substantial ${\text{V}}_{\text{TCA}}^{\text{g}}$ in cases of low glutamate-glutamine cycle rate.

To summarize, in addition to the proposed coupling of neuronal oxidative metabolism and neurotransmission, astrocytes increase their oxidative metabolism too, resulting in a large production of ATP. It is, therefore, important to investigate the exact fate of the ATP produced. In this context, the ATP produced in glia might notably support blood flow regulation (Zonta et al., 2003; Metea and Newman, 2006), neuronal activity modulation (Volterra and Meldolesi, 2005) and protection against oxidative stress (Borst and Elferink, 2002; Dringen and Hirrlinger, 2003). Glycogenolysis might moreover provide energy to support neurotransmission (i.e., release and uptake of glutamate; Sickmann et al., 2009).

## Functions of astrocytes in the brain

### Blood flow regulation

**Figure 6:**
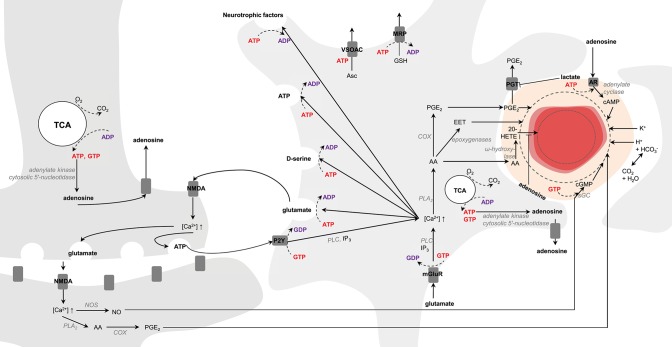
**Schematic representation of possible signaling pathways mediating neurovascular coupling**. Activation of glutamate receptors (mGluR, NMDA) triggers increases in Ca^2+^ concentration. Ca^2+^ increase upon mGluR activation is mediated by phospholipase C (PLC) and inositol 1,4,5-triphosphate (IP_3_). Arachidonic acid (AA) is then produced by phospholipase A_2_ (PLA_2_). In astrocytes AA can be converted either to prostaglandins E_2_ (PGE_2_) by cyclooxygenase (COX) or to epoxeicosatrienoic acids (EET) by epoxygenase for vasodilation. If AA is converted into 20-hydroxyeicosatetraenoic acid (20-HETE) by ω-hydroxylase, it will lead to vasoconstriction. In neurons, increase in Ca^2+^ concentration results in either PGE_2_ or nitric oxide (NO) production via NO synthase (NOS). In pericytes and smooth muscle cells NO interacts with the soluble guanylate cyclase (sGC) for cGMP- dependent vasodilation mechanisms. Neuronal release of vesicular ATP can activate the astrocytic purinergic receptors (P2Y) to raise intracellular Ca^2+^ concentration. Intracellular ATP can be converted to adenosine by adenylate kinase cytosolic 5′-nucleotidase. Intracellular adenosine can be transported by the nucleoside transporters to activate the adenosine receptors (AR) for cAMP-dependent vasodilation mechanisms via adenylate cyclase and inhibiting the vasoconstrictive effects of 20-HETE. Lactate can inhibit the astrocytic prostaglandin transporter (PGT)-mediated PGE-lactate exchange, increasing therefore extracellular PGE_2_ concentration. K^+^ and H^+^ ions, associated notably to action potentials and oxidative metabolism, respectively, can also modulate vasodilation. Astrocytes can modulate synaptic plasticity in releasing vesicles containing glutamate, D-serine, ATP and neurotrophic factors in an ATP-dependent manner. Glutathione is produced in astrocytes and can be released through multidrug resistance proteins (MRP) mediating ATP hydrolysis. Release of ascorbate mediate non-hydrolytic ATP binding to volume-sensitive organic osmolyte-anion channel (VSOAC) and is stimulated by glutamate. The left and right cells represent an astrocyte and a neuron, respectively. The difference in TCA cycle size reflects the fact that neurons are more oxidative than astrocytes. The red circle is a blood vessel, the surrounding darker layer represents the endothelial cells and the thicker outer line is the smooth muscle cells (or pericytes). Word in bold red and bold purple corresponds to energy-producing and consuming processes, respectively. Enzymes are in gray italic. The dashed line represents vasodilation and the dotted line vasoconstriction.

Brain vasculature is rich in arterioles and fine capillaries (Reina-De La Torre et al., 1998), and CBF regulation is mainly controlled by relaxation and constriction of these blood vessels (Attwell et al., 2010). In this context astrocytes and neurons are presumed to play a key role in modulating CBF to match energy demands. In neurons, upon NMDA activation intracellular Ca^2+^ concentration increases, which activate phospholipase A_2_ (PLA_2_) in the cytosol that then produces arachidonic acid (AA). In astrocytes, activation of mGluR by glutamate triggers the translocation of the α-subunit of the receptors to phospholipase C (PLC) mediating the conversion of GTP to GDP (Bockaert et al., 1993). Activated PLC cleaves membrane phosphatidylinositol 4,5-bisphosphate (PIP2) to diacylglycerol (DAG) and inositol 1,4,5-triphosphate (IP_3_) that triggers Ca^2+^ increase. In neurons, cyclooxygenase (COX) converts AA into prostaglandins E_2_ (PGE_2_) leading to vessel dilation (Wang et al., 2005). In astrocytes, AA can be converted either to PGE_2_ by COX (Zonta et al., 2003) or to epoxeicosatrienoic acids (EET) by epoxygenase, which diffuse through pericytes (Hamilton et al., 2010; or smooth muscle cells for arteries) for blood vessel dilation. If AA is converted into 20-hydroxyeicosatetraenoic acid (20-HETE) by ω-hydroxylase in pericytes, it will cause vasoconstriction (Metea and Newman, 2006). New line of evidence suggest moreover that the astrocytic production of PGE_2_ might be dependent on glutathione levels (Howarth et al., 2017, and references therein). In neurons activation of ionotropic glutamate receptors located on the post-synaptic zone (i.e., NMDA) also activates nitric oxide synthase (NOS), which in turn produces NO. Interaction of NO with soluble guanylate cyclase (sGC) triggers cGMP dependent vasodilation mechanisms (Laranjinha et al., 2012; Lourenço et al., 2014). Moreover, neuronal vesicular ATP can be released and act on astrocytic purinergic receptors (P2Y) to raise intracellular Ca^2+^ concentrations mediating the conversion of GTP to GDP and PLC activation (reviewed in Erb and Weisman, 2012; Bazargani and Attwell, 2016). Alternatively, ATP can also intracellularly be converted to adenosine by adenylate kinase cytosolic 5′-nucleotidase (reviewed in Iadecola, 2004). Intracellular adenosine can be released extracellularly by nucleoside transporters (Iliff et al., 2003), which can in turn act on adenosine receptors located on pericytes (Iliff et al., 2003; Gordon et al., 2008) and trigger the activation of adenylate cyclase (in mediating the conversion of GTP to GDP) that converts ATP to cAMP (Suzuki et al., 1988). Increase in cAMP leads to vasodilation and inhibits the vasoconstrictive effects of 20-HETE (Koehler et al., 2006; Figure 6).

Other lines of evidence suggest that the transfer of excess electrons from NADH to O_2_ by NADH oxidase (Wolin, 1996) can generate ${\text{O}}_{2}^{-}$, which raises intracellular Ca^2+^ levels (Ikebuchi et al., 1991) activating in turn NOS for NO production (Ido et al., 2001).

Two different dynamics of Ca^2+^ signaling have been proposed to initiate and sustain hemodynamic responses. First, brief (100 ms) Ca^2+^ responses in astrocytic end-feet, occurring downstream neuronal activation and scaling with the level of neuronal activity, triggers vessel dilation onset. Second, a slower and long lasting (seconds) Ca^2+^ elevation contributes to a prolonged blood vessel dilation (Lind et al., 2013). Yet, the observation that astrocytic Ca^2+^ can lag a few seconds arteriolar dilation supports the hypothesis that astrocytic Ca^2+^-dependent mechanisms may not be a prerequisite for CBF response initiation (Nizar et al., 2013). Local CBF response could be immediately regulated by fast (400 ms) feed-forward mechanisms directly related to neuronal activity (e.g., neuronal NO production (Buerk et al., 2003), action potential-associated K^+^ current (Paulson and Newman, 1987), neuronal arachidonic pathway activation Zonta et al., 2003; Metea and Newman, 2006), rather than feedback mechanisms associated with metabolism (e.g., lactate, astrocytic mGluR-related signaling, ATP-derived adenosine signaling), that probably occur at longer time scales (seconds) to match CBF with energy demands (discussed in Buxton, 2010). In this context the fact that the CBF/CMR_O2_ ratio varies between brain regions, as well with stimulus frequency (discussed in Buxton, 2010) and length (Lin et al., 2009), further suggests regulation of this ratio by notably neuronal activity-associated mechanisms (discussed in Buxton, 2010). Recent data further address this controversy in suggesting Ca^2+^-dependent signaling for modulation of capillary but not arteriolar diameter (Biesecker et al., 2016). Because CO_2_, one of the end products of oxidative metabolism, can diffuse out of the cells and is in rapid equilibrium with ${\text{HCO}}_{3}^{-}$, extracellular H^+^ ions can also locally contribute to CBF regulation (Kuschinsky and Wahl, 1978).

### Neuronal activity modulation and synaptic plasticity

Although astrocytes do not generate action potentials *per se* they can actively modulate synaptic transmission and neuronal synchronization in mediating notably the release of vesicles-containing neurotransmitters and neuromodulators, such as glutamate, ATP, adenosine, and D-serine. Glutamate-containing microvesicles are present in astrocytes and upon Ca^2+^ signaling glutamate molecules are released and can target post-synaptic receptors (i.e., NMDA, AMPA, mGluR, and kainate) to fine tune firing threshold (Volterra and Meldolesi, 2005). In the extracellular space ATP can be converted to adenosine by the dephosphorylating action of the ectonucleotidase anchored at the plasma membrane (Joseph et al., 2003). Adenosine can activate adenosine receptors and therefore modulate neuronal activity by triggering K^+^ efflux (e.g., Newman, 2003), as well as intermediary metabolism (Haberg et al., 2000; Hammer et al., 2001; Duarte et al., 2016) and blood flow (Blood et al., 2003; Iliff et al., 2003; Gordon et al., 2008). D-serine that can be released from astrocytes was also shown to modulate electrical neurotransmission by acting at the glycine binding site of NMDA receptor (Stevens et al., 2003). Moreover, the production of neurotrophic factors was shown to promote the formation and the function of synapses (Pfrieger and Barres, 1997), and to regulate intracellular Ca^2+^ homeostasis upon stimulation of the glutamate receptors and thus to preserve the activity of the mitochondrial electrochemical gradient and therefore energy metabolism (El Idrissi and Trenkner, 1999). Glutamate, D-serine, ATP and neurotrophic factors are notably released exocytotically, mediating Ca^2+^ signaling and ATP-dependent soluble *N*-ethylmaleimide-sensitive factor activating protein receptor (SNARE) disassembly (Goda, 1997; Parpura and Zorec, 2010), resulting in energy consumption (Figure 6). The delivery of these vesicles to the plasma membrane involves cytoskeleton assembly/disassembly (Potokar et al., 2007) requiring ATP hydrolysis (Korn et al., 1987; Le Clainche et al., 2003). Interestingly, the number of astrocytic processes, as well as their contact with active synapses, are stimulated by extracellular glutamate and also involve actin-dependent mechanisms (Cornell-Bell et al., 1990) requiring ATP hydrolysis (Korn et al., 1987; Le Clainche et al., 2003).

### Protection against oxidative stress

The use of NADH through the electron transport chain for ATP production and mitochondrial Ca^2+^ influx results in reactive oxygen species (ROS) production (Boveris and Chance, 1973; and reviewed in Görlach et al., 2015). Yet, the cooperative action of astrocytes in culture was shown to protect neurons against ROS toxicity (Desagher et al., 1996), and astrocytes express larger amounts of antioxidant molecules and ROS-detoxifying enzymes than neurons (Makar et al., 1994). The thiol group of the glutathione molecule acts as an important electron donor. While both neurons and astrocytes synthesize glutathione, neuronal glutathione levels are higher in the presence of astrocytes (Dringen et al., 1999), probably because of shuttling of cysteine-glycine (glutathione precursor) from astrocytes to neurons (Dringen and Hirrlinger, 2003). Glutathione transport across cells is notably mediated by multidrug resistance proteins (MRP) that belong to the subgroup ABCC of the ATP-binding cassette transporters, which mediate passage via ATP hydrolysis (Borst and Elferink, 2002; Dringen and Hirrlinger, 2003; Figure 5). Activation of astrocytic glutamate receptors was shown to translocate nuclear factor-erythroid 2-realted factor-2 (Nrf2) (present in lower concentrations in neurons) into the nucleus and to trigger the expression of antioxidant genes, notably related to glutathione metabolism (Jimenez-Blasco et al., 2015). Astrocytes synthesize large amount of hydrogen sulfide, which was demonstrated to not only have neuroprotective properties (Lee et al., 2009), but also act as neuromodulator in enhancing NMDA responses (Abe and Kimura, 1996) and modulating glial Ca^2+^ waves (Nagai et al., 2004).

Ascorbate is also another important antioxidant anion in the brain and glutamate was demonstrated to stimulate its release from astrocytes (Wilson et al., 2000), suggesting an essential protecting role of the ascorbate flux from astrocytes to neurons during synaptic activity (Acuña et al., 2013). Astrocytes are responsible for the recycling of the neuronal extracellularly released dehydroascorbic acid (the oxidized form of ascorbate) into ascorbate, which can be exported to neurons (Covarrubias-Pinto et al., 2015). Extracellular transport of ascorbate from astrocytes is believed to be mediated by volume-sensitive organic osmolyte-anion channel (VSOAC) that requires non-hydrolytic ATP binding (Jackson et al., 1994; Covarrubias-Pinto et al., 2015; Figure 6). Considering the fact that efficacy of the mechanisms stimulated by astrocytic glutamate uptake depends on the density of the transporters at the plasma membrane (Robinson, 2002), efficient trafficking of EAAT2-containing vesicles and exocytosis must moreover take place (Stenovec et al., 2008).

### Glycogenolysis

Glycogen constitutes a glucose storage in the form of highly branched polysaccharide molecules (Preiss and Walsh, 1981) found in high concentrations in the liver and in skeletal muscle, although smaller but significant levels (6–8 μmol/g) are also estimated in the human brain (Oz et al., 2015). Glycogenesis (glycogen production from glucose 1-phosphate by glycogen synthase) and glycogenolysis (glycogen breakdown to glucose 6-phosphate by the combined action of glycogen phosphorylase and phosphoglucomutase) mainly occurs in astrocytes (Dringen et al., 1993; Figure 1), and the latter can be stimulated by extracellular glutamate and K^+^ (Hertz et al., 2015). In line with this, glycogen levels were found to increase with anesthesia (Morgenthaler et al., 2006) and decrease during somatosensory (Swanson et al., 1992) and visual (Dienel et al., 2007a) stimulation. However, no change in brain glycogen level was measured during visual stimulation in humans (Oz et al., 2007). While glycogen-derived lactate has been demonstrated to have a pivotal role in memory formation and consolidation (Gibbs and Hertz, 2005; Suzuki et al., 2011; Boury-Jamot et al., 2016), learning mechanism and synaptic strength (Duran et al., 2013), and neuronal function (Tekkök et al., 2005), the role of glycogen is unlikely limited to fuel neuronal metabolism. Recently, astrocytic glycogenolysis was shown to provide energy to sustain glutamatergic neurotransmission (i.e., glutamate uptake and release; Sickmann et al., 2009). Glycogen might act as a substrate for *de novo* formation of glutamate (Gibbs et al., 2007) and glycogen-derived energy might be required over glucose-derived energy for pyruvate carboxylation (Sickmann et al., 2012), suggesting that astrocytic glycogen metabolism might be crucial to maintain proper brain function.

## Conclusion

While the essential role of astrocytes to cerebral function is now widely accepted, quantitative assessment of their actual contribution to energy metabolism has been missing, notably because the methodologies did not allow differentiating between neurons and astrocytes. Direct ^13^C MRS along with advanced metabolic modeling can provide measurements of both neuronal and glial metabolism in specific brain regions and under various activation states. In this context, new data indicate that the rate of astrocytic metabolism is about half of that in neurons, and can be activated by sensory stimulation and that the astrocytic response amplitude can be, in absolute terms, as large as in neurons, suggesting that the changes in ATP requirements associated with the glutamate-glutamine cycle are coupled with the ATP produced by glucose oxidation in both compartments.

Increase in neuronal metabolism likely supports neurotransmission-associated functions, such as restoration of ion gradients caused by action potentials, post-synaptic currents, and transport of glutamate into vesicles. Adaptation of glial metabolism also provides energy for neurotransmission besides housekeeping tasks, likely fueling the production and action of modulators of neuronal activity and of synaptic plasticity, supply of antioxidant molecules and neurotrophic factors that are necessary for adequate brain function, and regulation of blood flow and volume. Astrocytes are moreover important source of glycogen that can be used specifically for neurotransmission support.

Progress in MR detection methods of ^1^H and non-^1^H nuclei is a promising direction for more detailed and complete metabolic dataset acquisition. While this provides insights into cellular function *in vivo*, it also requires improvement of current metabolic models describing best energy metabolism. Simultaneous acquisition of other types of data, such as electrical activity and blood flow, might contribute to more precise characterization of the coupling between brain function and energy metabolism by MRS.

## Author contributions

SS and JD wrote the manuscript. RG revised the manuscript.

### Conflict of interest statement

The authors declare that the research was conducted in the absence of any commercial or financial relationships that could be construed as a potential conflict of interest.

## Abbreviations

20-HETE: 20-hydroxyeicosatetraenoic acid
AA: arachidonic acid
ADP: adenosine 5′-diphosphate
AMPA: α-amino-3-hydroxyl-5-methyl-4-isoxazole-propionate
ANLS: astrocyte-neuron lactate shuttle
AST: aspartate transaminase
ATP: adenosine 5′-triphosphate
BBB: blood-brain barrier
BOLD: blood oxygenation level-dependent
cAMP: cyclic adenosine monophosphate
CBF: cerebral blood flow
CBV: cerebral blood volume
CMR_glc_: cerebral metabolic rate of glucose
CMRO_2_: cerebral metabolic rate of oxygen
COX: cyclooxygenase
DNP: dynamic nuclear polarization
EAAT: excitatory amino acid transporters
EET: epoxeicosatrienoic acid
FADH_2_: flavin adenine dinucleotide (reduced form)
FE: fractional enrichment
fMRI: functional magnetic resonance imaging
fMRS: functional magnetic resonance spectroscopy
FRET: fluorescence resonance energy transfer
GDH: glutamate dehydrogenase
GDP: guanosine diphosphate
GLS: glutaminase
GLUT: glucose carrier
GS: glutamine synthetase
GTP: guanosine triphosphate
IP_3_: inositol 1,4,5-triphosphate
K_t_: apparent Michaelis constant of glucose transport
MCT: monocarboxylate transporters
ME: malic enzyme
mGluR: metabotropic glutamate receptor
MR: magnetic resonance
MRP: multidrug resistance proteins
MRS: magnetic resonance spectroscopy
NADH: nicotinamide adenine dinucleotide (reduced form)
NMDA: *N*-methyl-D-aspartate
NO: nitric oxide
NOS: nitric oxide synthase
Nrf2: nuclear factor-erythroid 2-realted factor-2
OAA: oxaloacetate
OG: 2-oxoglutarate
P2Y: purinergic receptors
PC: pyruvate carboxylase
PDH: pyruvate dehydrogenase complex
PEPCK: phosphoenolpyruvate carboxykinase
PET: positron emission tomography
PFK: phosphofructokinase-1
PFKFB3: fructose-2,6-bisphosphatase-3
PGE_2_: prostaglandins E_2_
PK: pyruvate kinase
PLA_2_: phospholipase A_2_
PLC: phospholipase C
SA: system A transporter
sGC: soluble guanylate cyclase
SLC2: solute carrier family 2
SN: system N transporter
SNARE: soluble *N*-ethylmaleimide-sensitive factor activating protein receptor
CA: tricarboxylic acid cycle
T_max_: apparent maximum transport rate of glucose transport
VGLUT: vesicular glutamate transporter
VSOAC: volume-sensitive organic osmolyte-anion channel.
